# iPSC-derived hepatocytes generated from NASH donors provide a valuable platform for disease modeling and drug discovery

**DOI:** 10.1242/bio.055087

**Published:** 2020-12-16

**Authors:** Igor Gurevich, Sarah A. Burton, Christie Munn, Makiko Ohshima, Madelyn E. Goedland, Katherine Czysz, Deepika Rajesh

**Affiliations:** Life Science R&D Division, FUJIFILM Cellular Dynamics, Inc., 525 Science Drive, Madison, WI 53711, USA

**Keywords:** Hepatocyte, Stem cell differentiation, Fatty liver disease, NASH, Co-culture

## Abstract

Non-alcoholic fatty liver disease (NAFLD) affects 30–40% of adults and 10% of children in the US. About 20% of people with NAFLD develop non-alcoholic steatohepatitis (NASH), which may lead to cirrhosis and liver cancer, and is projected to be a leading cause of liver transplantation in the near future. Human induced pluripotent stem cells (iPSC) from NASH patients are useful for generating a large number of hepatocytes for NASH modeling applications and identification of potential drug targets. We developed a novel defined *in vitro* differentiation process to generate cryopreservable hepatocytes using an iPSC panel of NASH donors and apparently healthy normal (AHN) controls. iPSC-derived hepatocytes displayed stage specific phenotypic markers, hepatocyte morphology, with bile canaliculi. Importantly, both fresh and cryopreserved definitive endoderm and hepatoblasts successfully differentiated to pure and functional hepatocytes with increased CYP3A4 activity in response to rifampicin and lipid accumulation upon fatty acid (FA) treatment. End-stage hepatocytes integrated into three-dimensional (3D) liver organoids and demonstrated increased levels of albumin secretion compared to aggregates consisting of hepatocytes alone. End-stage hepatocytes derived from NASH donors demonstrated spontaneous lipidosis without FA supplementation, recapitulating a feature of NASH hepatocytes *in vivo*. Cryopreserved hepatocytes generated by this protocol across multiple donors will provide a critical cell source to facilitate the fundamental understanding of NAFLD/NASH biology and potential high throughput screening applications for preclinical evaluation of therapeutic targets.

## Abstract (precis)

**Summary:** We describe the development and validation of a hepatocyte differentiation protocol using iPSCs from a panel of NASH donors and healthy controls.

## INTRODUCTION

Non-alcoholic fatty liver disease (NAFLD) is a multisystem disease, associated with chronic liver disease as well as affecting extra-hepatic organs and regulatory pathways. About one-fifth of NAFLD patients go on to develop non-alcoholic steatohepatitis (NASH), the most severe form of NAFLD (Spengler and Loomba, 2015). NASH is characterized by increased lipid accumulation in hepatocytes, coupled with liver fibrosis and hepatocyte ballooning (Takahashi and Fukusato, 2014). While often asymptomatic, NASH can progress to cirrhosis of the liver and liver cancer and is projected to overtake hepatitis C as the leading cause of liver transplantation in the near future (Parikh et al., 2019). NAFLD and NASH are associated with environmental factors such as diet and level of physical activity, and metabolic disorders such as type 2 diabetes are often comorbid with NASH. Genetic risk factors, such as the I148M polymorphism in *PNPLA* gene (Romeo et al., 2008) are also associated with increased susceptibility. It is clear that the etiology of NAFLD and NASH is complex and involves various factors, of which the interplay is still poorly understood.

Significant weight loss through lifestyle modification (Vilar-Gomez et al., 2015) or bariatric surgery (Talavera-Urquijo et al., 2020) have been shown to be helpful in resolution of NASH. There has also been considerable research activity aimed at developing a pharmacologic intervention against NASH with several compounds currently in clinical trials. These compounds have diverse mechanisms of action that generally focus on metabolic pathways that are disrupted in the disease state (Esler and Bence, 2019).

A strong interest in developing therapies for NAFLD and NASH has created an impetus for generating *in vitro* models to study NASH development and to evaluate prospective drugs. Human induced pluripotent stem cells (hiPSC) with their unlimited proliferative capacity and ability to differentiate into different cell types provide a potential for generating large batches of cryopreserved end stage lineages for *in vitro* disease modelling applications. Indeed, hiPSC derived hepatocytes have been generated to mimic different aspects of fatty liver disease (Parafati et al., 2018). In addition to hepatocytes, the liver also contains Kupffer cells, hepatic stellate cells, and sinusoidal endothelial cells. An optimal *in vitro* model should include multiple cell type approaches to recapitulate the liver complexity for disease modeling (Underhill and Khetani, 2019).

NASH patient-derived cells can serve as a valuable tool in understanding the disease progression and drug development. This study included iPSC lines derived from donors with NASH along with apparently healthy normal (AHN) controls to develop a novel hepatocyte differentiation protocol. This protocol is robust, i.e. it performed consistently well across iPSCs from multiple donor backgrounds and yielded cryopreservable hepatocytes with a high purity of hepatic markers that recapitulated other features of hepatocyte functionality including drug metabolism and formation of bile canaliculi. Hepatocytes produced by this protocol were amenable to co-culture with other liver relevant cell types: macrophages, mesenchymal stem cells, and endothelial cells. When exposed to fatty acids (FA), hepatocytes produced by this protocol demonstrated dose dependent intracellular lipid accumulation. While no difference in hepatic differentiation capacity and functional assays between cells from AHN and NASH iPSC lines is observed, end-stage hepatocytes from NASH donors revealed higher levels of lipid accumulation than those from AHN controls even in the absence of added FA, thus displaying a hallmark of NASH hepatocytes *in vivo*.

## RESULTS

### Development of hepatocyte differentiation protocol

**Fig. 1.**
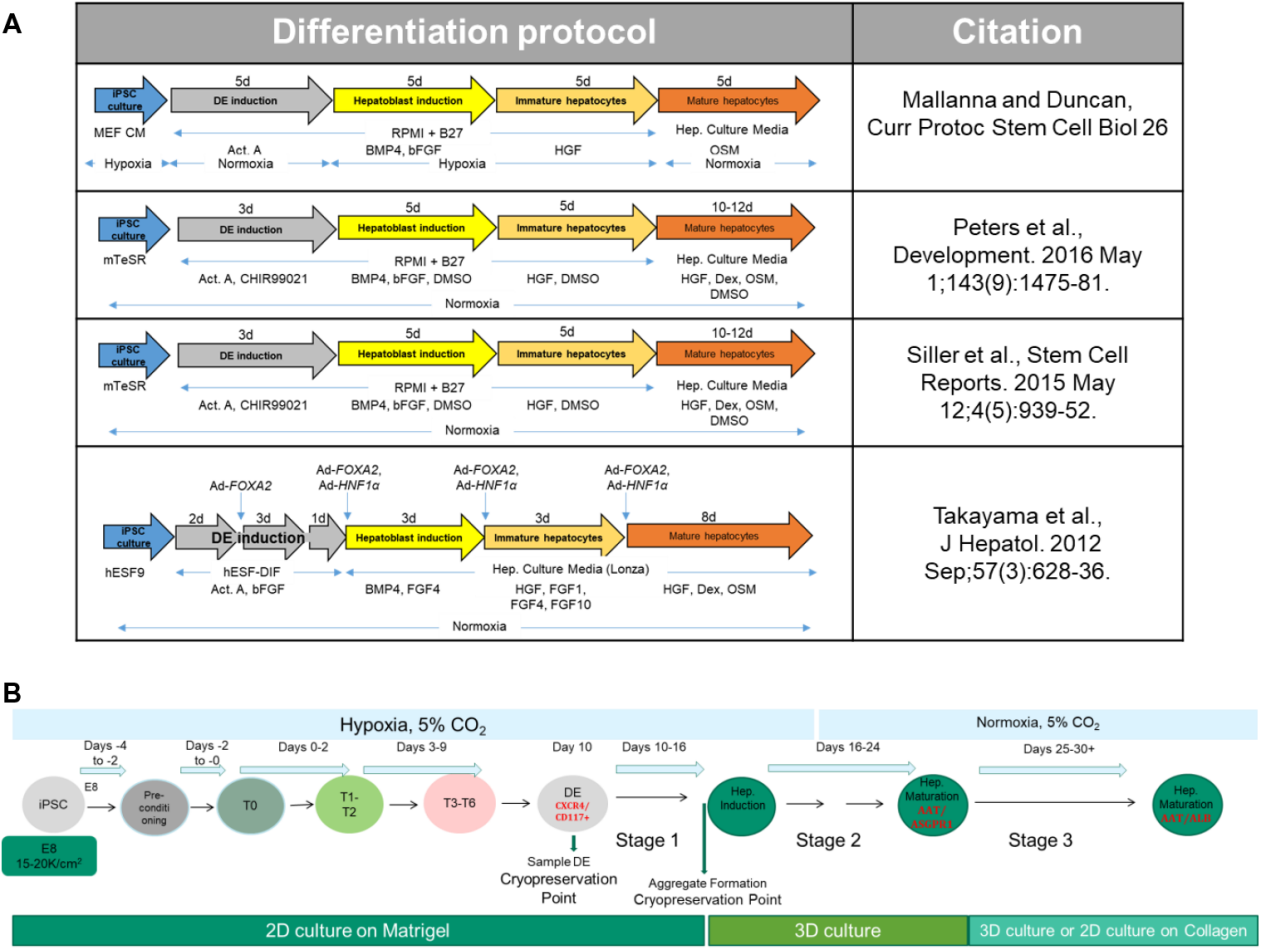
**Development of hepatocyte differentiation protocol.** (A) Representative published protocols for differentiation of hiPSCs into hepatocytes. MEF CM, mouse embryonic fibroblast conditioned media; Hep. Culture Media, hepatocyte culture media (Lonza); hESF9, defined serum free media for culture of human embryonic stem cells (Furue et al., 2008); hESF-DIF, human ESC differentiation media (Cell Science & Technology Institute, Inc.); Ad-*FOXA2* and Ad-*HNF1α*, adenoviral vectors for transduction of transcription factors FOXA2 and HNF1α. (B) Schematic of hepatocyte differentiation process. iPSCs from AHN and NASH donors were maintained in E8/Matrigel and acclimatized to hypoxic conditions. To initiate differentiation, iPSCs were expanded and preconditioned prior to starting DE differentiation for 10 days. The purity of DE cultures was assessed and DE cells were transitioned to hepatoblasts (Stage 1). At the end of this stage of differentiation, the cells were detached to form aggregates and differentiated further to generate mature hepatocytes. Cells can be cryopreserved at indicated points during the differentiation process, and successfully differentiated to live end-stage hepatocytes.

Episomally reprogrammed iPSCs generated from healthy (AHN) donors and NASH patients were used to develop the differentiation protocol described here. The protocol evolved from several published (Mallanna and Duncan, 2013; Peters et al., 2016; Siller et al., 2015; Takayama et al., 2012) hepatocyte differentiation protocols (Fig. 1A) by examining and modifying media compositions and culture methods at each stage of differentiation. Throughout the development of the protocol, improvements were adapted to increase the consistency of the process utilizing iPSCs from different donor backgrounds, healthy or diseased. The different stages of the finalized hepatocyte differentiation process are captured in Fig. 1B.

### Preconditioning with CHIR enhanced the generation of definitive endoderm (DE) cells across iPSC lines

**Fig. 2.**
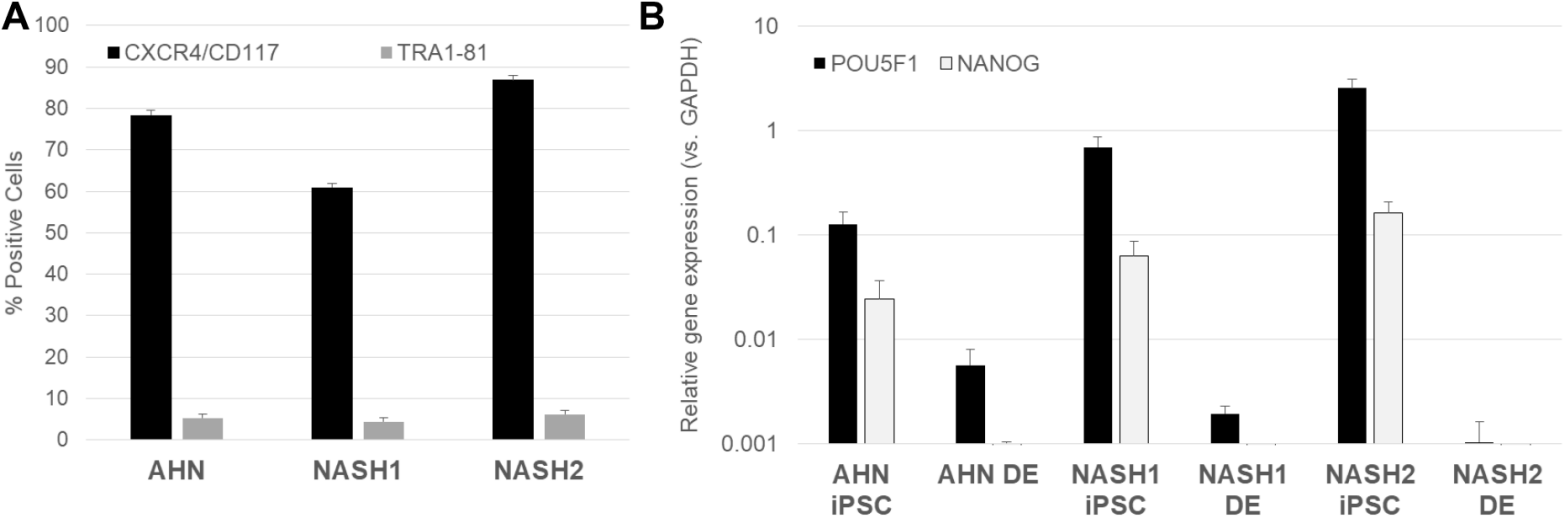
**Exit from pluripotency and DE induction.** (A) Flow cytometry analysis for quantification of DE markers CXCR4 and CD117 as well as expression of pluripotency marker TRA1-81 at the end of DE induction in lines from AHN and NASH1 and NASH2 derived IPSCs. (B) Quantification of pluripotency genes *POU5F1* and *NANOG* between iPSCs and DE derived from ANH and NASH donors by qPCR analysis. The graphs denote average values±s.e. from three differentiation runs.

The first phase of differentiation process involved generation of DE. iPSCs derived from AHN and NASH specific donors consistently yielded pure population of DE cells defined by the co-expression of CXCR4 and CD117 (Fig. 2A). Efficient DE induction was coupled with the decline of pluripotency markers OCT4, NANOG, and TRA1-81 (Fig. 2A,B).

**Fig. 3.**
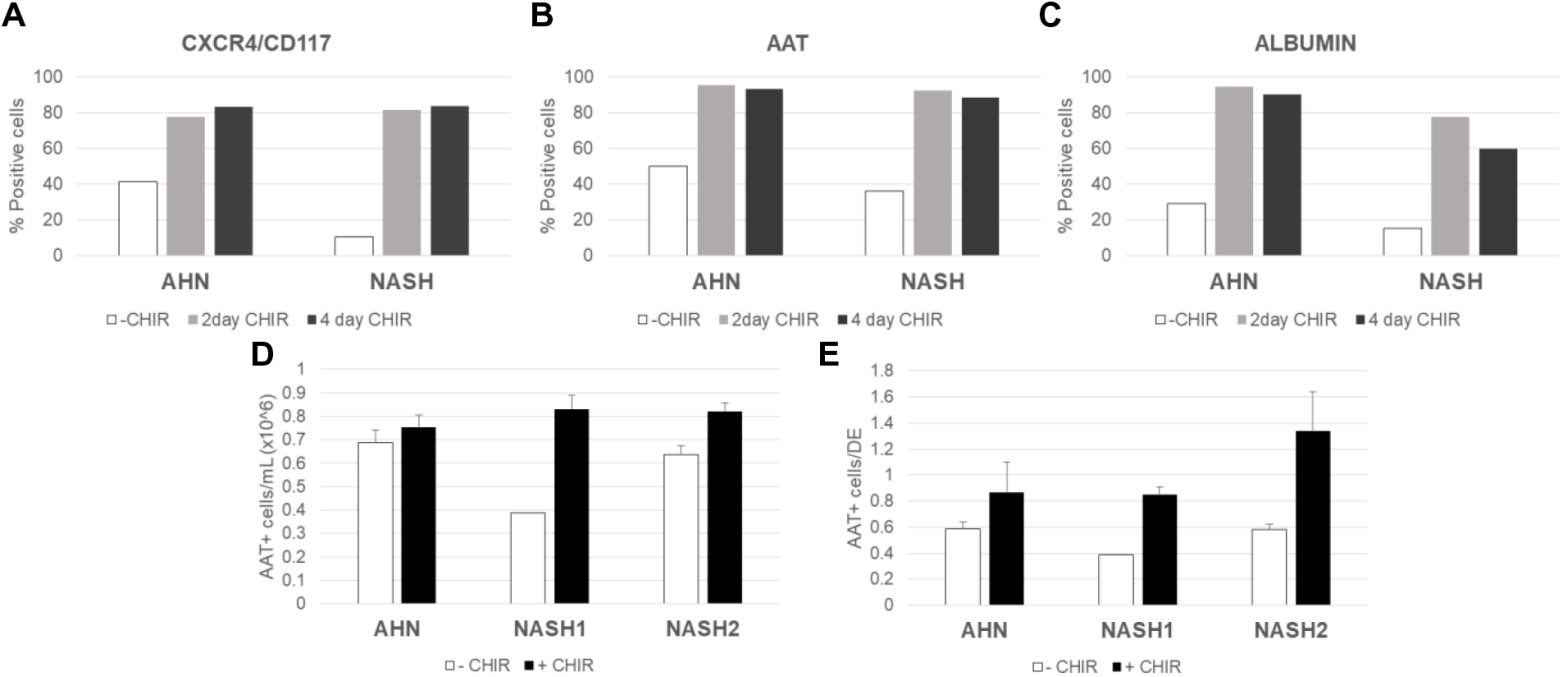
**Effects of CHIR99021 use in the preconditioning stage and Stage 2 of the differentiation process.** (A) Quantification of co-expression of DE markers CXCR4 and CD117 at the end of DE induction in lines from AHN and NASH donors without preconditioning (−CHIR) or with preconditioning for 2 (2-day CHIR) or 4 (4-day CHIR) days by flow cytometry. The data includes an average of duplicate samples. (B) Quantification of AAT expression at the end of process in lines from AHN and NASH donors without preconditioning (-CHIR) or with CHIR99021 preconditioning for 2 (2-day CHIR) or 4 (4-day CHIR) days by flow cytometry analysis. The data includes an average of duplicate samples. (C) Quantification of albumin expression at the end of process in lines from AHN and NASH donors without preconditioning (-CHIR) or with CHIR99021 preconditioning for 2 (2-day CHIR) or 4 (4-day CHIR) days by flow cytometry. The data includes an average of duplicate samples. (D) Yields of AAT+ hepatocytes/ml of cell culture (culture volumes were kept equal between conditions), and (E) efficiency, defined as ratio of AAT+ cells at end of Stage 2 to number of cells at the end of DE induction, in lines from AHN and NASH donors cultured in the absence (-CHIR) or presence (+CHIR) of CHIR99021 during Stage 2 of differentiation. AAT purity quantified by flow cytometry. The graphs denote average values±s.e. from three independent experiments.

Preconditioning of iPSCs with CHIR99021, a GSK3 inhibitor, either for 2 or 4 days prior to initiating DE differentiation, enhanced the efficiency of conversion of iPSC to DE cells (Fig. 3A), which further resulted in a high level of expression of both alpha-1 antitrypsin (AAT) and albumin in the end-stage hepatocyte cultures (Fig. 3B,C). There were no significant differences in outcomes between the cells preconditioned with CHIR99021 for 2 versus 4 days and thus, a 2-day preconditioning step was adapted as a routine step in the protocol.

### CHIR supplementation improved the efficiency of hepatocyte generation

A beneficial effect of CHIR99021 supplementation was noted during the conversion of hepatoblasts to hepatocytes during Stage 2 of the differentiation process. Incorporation of CHIR99021 during Stage 2 resulted in a pronounced increase in the overall cell number resulting in higher hepatocyte yields (Fig. 3D), improving the overall process efficiency – the ratio of AAT+ cells per number of cells at the end of DE induction – across multiple donor lines (Fig. 3E).

### End stage cells exhibited hepatic phenotypic characteristics

**Fig. 4.**
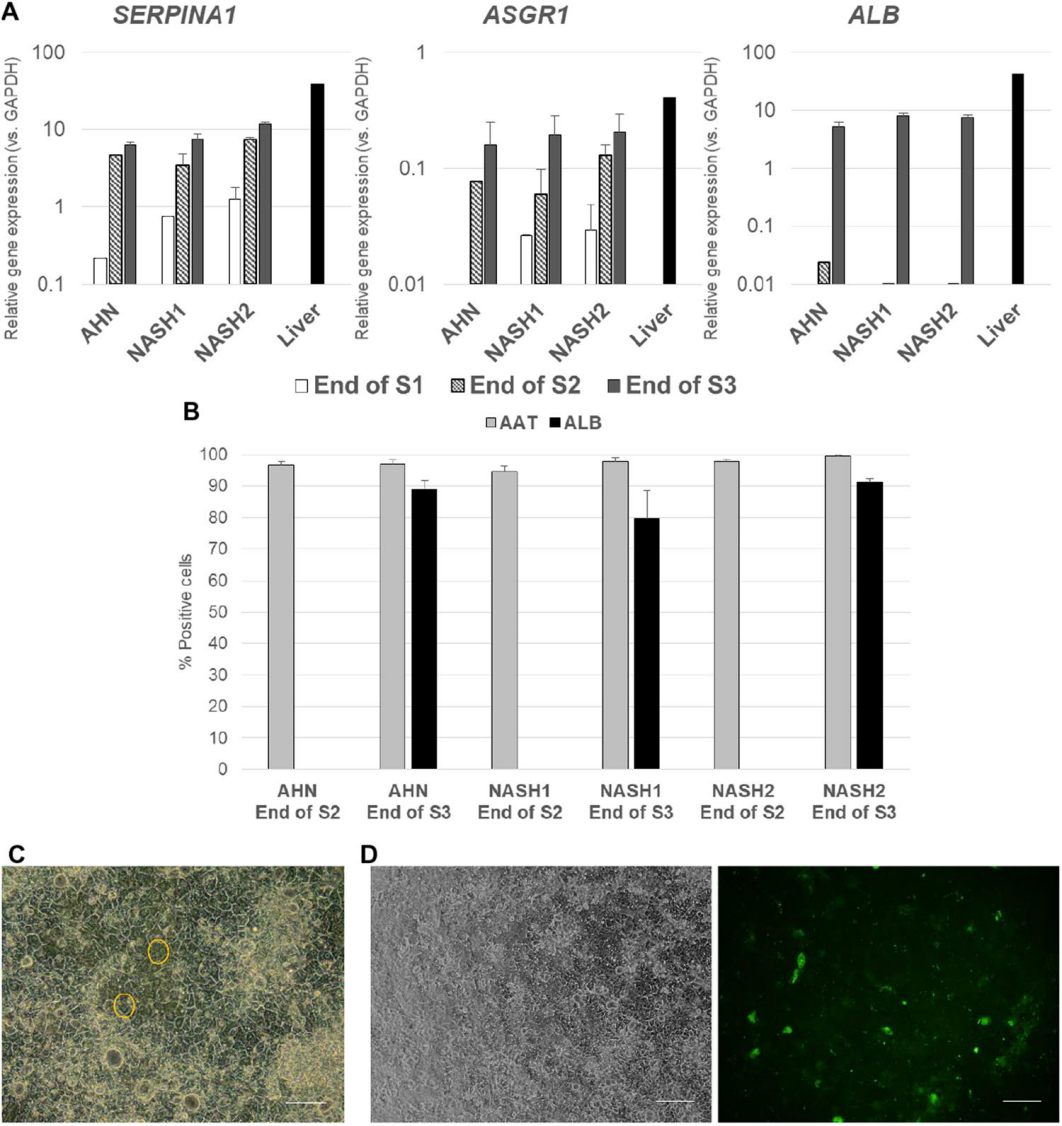
**Acquisition of hepatic markers in differentiating hepatocytes.** (A) Quantification of *SERPINA1*, *ASGR1*, and *ALB* gene expression in lines from AHN and NASH donors at end of Stages 1, 2, and 3 of hepatocyte differentiation along with total RNA from adult human liver by qPCR analysis. The graphs denote average values±s.e. for each condition. (B) Quantification of AAT and albumin (ALB) expression in AHN and NASH iPSC derived hepatocytes at the end of Stages 2 and 3 of hepatocyte differentiation. The graph denotes average values±s.e. from three independent experiments. (C) Representative image of the hepatocytes taken 7 days after plating onto collagen plates at the end of Stage 2 and culturing in Stage 3 media. Binucleate cells, a key hepatocyte feature, are highlighted in yellow. (D) Representative images of the hepatocytes taken 7 days after plating onto collagen plates at the end of Stage 2, culturing in Stage 3 media and staining with CDFDA to visualize bile canaliculi (left, bright field; right, CDFDA staining). Scale bars: 200 µm.

As the iPSCs progressed through different stages of the hepatocyte differentiation process, an increase in the expression level of hepatic markers *SERPINA*, *ASGR1*, and *ALB* – genes encoding AAT, asiaglycoprotein 1, and albumin – was quantified. The level of expression approached levels detected in adult human liver (Fig. 4A). End-stage hepatocyte cultures revealed a high purity (nearly 100%) of AAT-positive cells with half or more cells co-expressing albumin (Fig. 4B). When placed on Collagen I coated plates at the end of Stage 2 and cultured in Stage 3 maturation media, the cultures exhibited cobblestone morphology with the presence of binucleate cells (Grizzi and Chiriva-Internati, 2007), microscopic feature typical of hepatocytes and formed bile canaliculi detected by CDFDA staining (Fig. 4C,D).

**Fig. 5.**
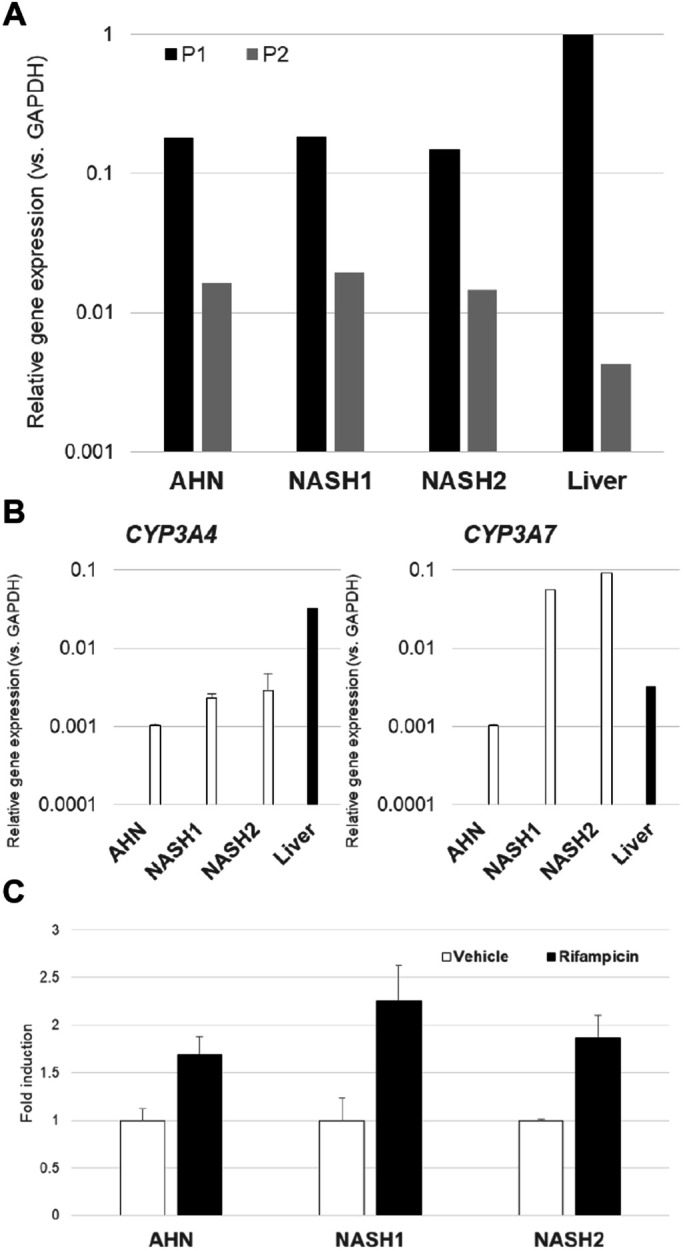
**HNF4α and cytochrome P450 expression and activity in end of process hepatocytes.** Quantification of (A) P1 and P2 transcripts of *HNF4A* gene and (B) *CYP3A4* and *CYP3A7* genes in end of process hepatocytes differentiated from AHN and NASH donor iPSC by qPCR analysis. The graphs denote average values±standard error from three independent experiments. The graphs denote average values±standard error from three independent experiments alongside total RNA from adult human liver. (C) Induction of CYP3A4 activity by rifampicin in end stage hepatocytes differentiated from AHN and NASH donor iPSCs. The graphs denote average values±standard error from four to six biological replicates per condition.

In order to further assess the level of hepatic maturity, the expression profile of the nuclear receptor HNF4α was quantified. This receptor is a key regulator of numerous hepatic processes and its expression is necessary for liver development. The gene encoding HNF4α, *HNF4A*, is under transcriptional control of two distinct promoters, P1 and P2. P1 transcripts are characteristic of more mature hepatocytes while P2 transcripts are characteristic of fetal hepatocytes (Babeu and Boudreau, 2014; Chavalit et al., 2013). P1 transcripts were predominantly detected in adult liver RNA samples and end stage hepatocytes generated by the current differentiation protocol (Fig. 5A).

Xenobiotic metabolism is an important hallmark of hepatocytes and the profile of the enzymes responsible for xenobiotic metabolism changes between fetal, neonatal, and adult. A well-known indicator of hepatocyte maturation is the switch in the dominant isoform of CYP3A enzyme from CYP3A7 in the fetal and neonatal hepatocytes to CYP3A4 shortly after birth (Lacroix et al., 1997). End stage iPSC derived hepatocytes exhibited a higher level of *CYP3A7* expression than *CYP3A4* levels (Fig. 5B). Although the level of *CYP3A4* expression was tenfold less than that in adult human liver, the cells from both AHN and NASH donors demonstrated a ∼twofold rifampicin mediated induction of CYP3A4 activity (Fig. 5C).

Taken together, the expression profiles of *HNF4A* and *CYP3A* indicate that the end stage hepatocytes generated by this protocol are at an intermediate level of maturity between fetal and adult hepatocytes.

### Hepatocyte recovery from cryopreservation

**Fig. 6.**
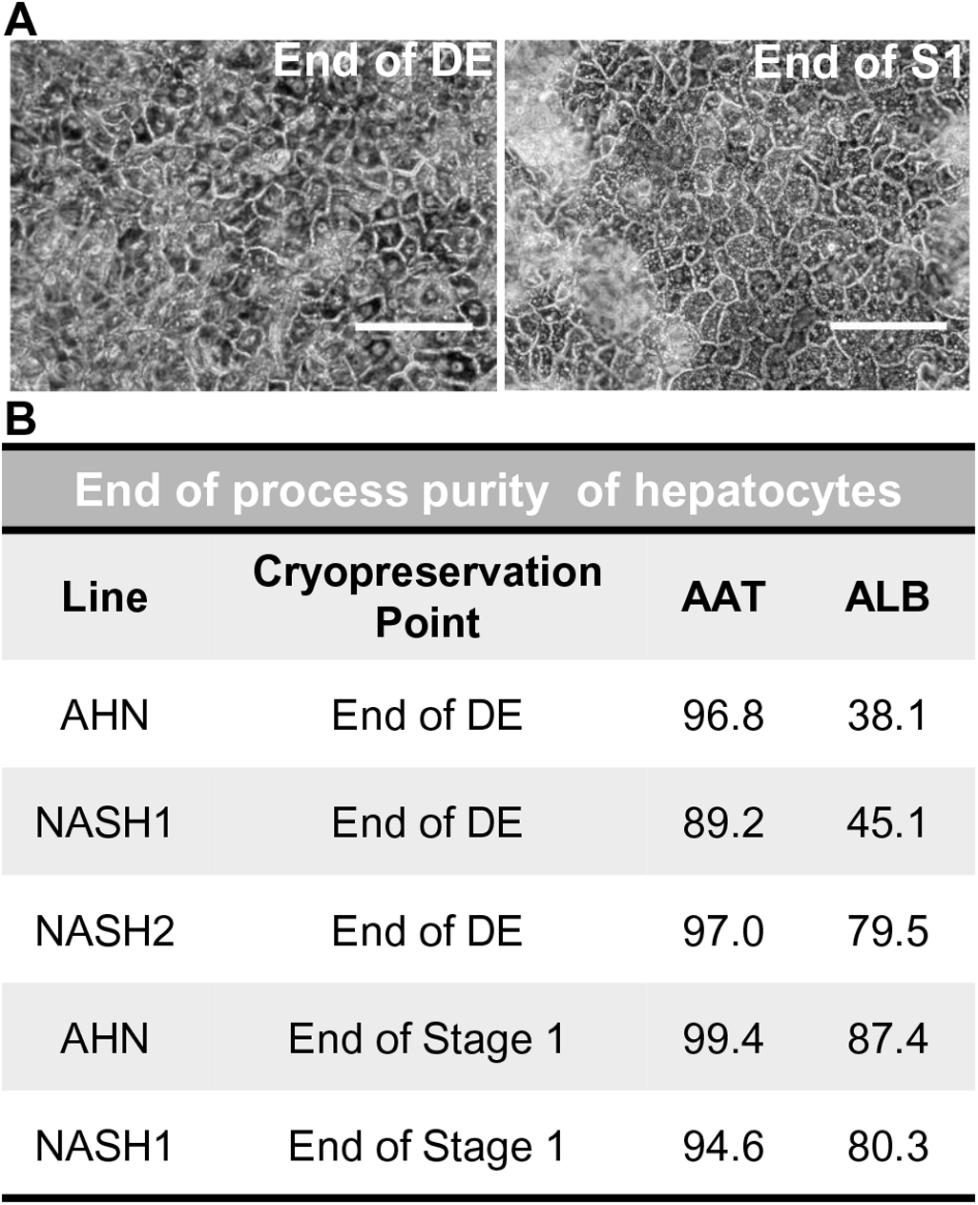
**Morphology and hepatic protein purity of hepatocytes after recovery from cryopreservation.** (A) Representative images of Stage 3 NASH hepatocytes differentiated from cryopreserved DE (left) or end of Stage 1 (right). Cells were thawed, differentiated through the end of Stage 2, seeded onto Collagen I coated plates and cultured further for 8 days in Stage 3 media. Scale bars: 100 µm. (B) Summary of the quantification of hepatic specific markers AAT and ALB expression in end-stage hepatocytes derived from cells cryopreserved at the end of DE or end of Stage 1 by flow cytometry analysis. In all cases, cryopreserved cells were thawed and placed in differentiation to generate end-stage hepatocytes.

The ability to cryopreserve iPSC derived hepatocytes greatly increases their experimental utility. To this end, cryopreservation of the cells was attempted at various time points during the differentiation process. Although cells cryopreserved at the very last step of differentiation exhibited poor recovery, cells were amenable to cryopreservation at earlier stages of the process. Cells frozen at the end of DE or Stage 1 recovered well after cryopreservation and successfully differentiated to end stage hepatocytes. The cells typically exhibited >80% viability at thaw and typical hepatocyte morphology when plated onto collagen I coated vessels (Fig. 6A). Moreover, they routinely progressed to end stage pure hepatocytes with high AAT and albumin levels similar to non-cryopreserved or fresh end stage cultures (Fig. 6B). The cells generated at the end of Stage 2 of the differentiation were only moderately amenable to cryopreservation across different donor iPSCs. Hence, intermediate cell populations cryopreserved at the end of DE or Stage 1 differentiation offered a more consistent option for cryopreservation with AHN and disease specific iPSC lines.

### Formation of liver organoids

The liver is composed of epithelial cells (hepatocytes and cholangiocytes) that work together with stromal, endothelial cells, mesenchymal cells and Kupffer cells to perform crucial metabolic functions (Cotovio and Fernandes, 2020; Lee et al., 2020). Organoid cultures recapitulating this complexity have emerged as a useful *in vitro* system to model tissue behavior in a dish.

**Fig. 7.**
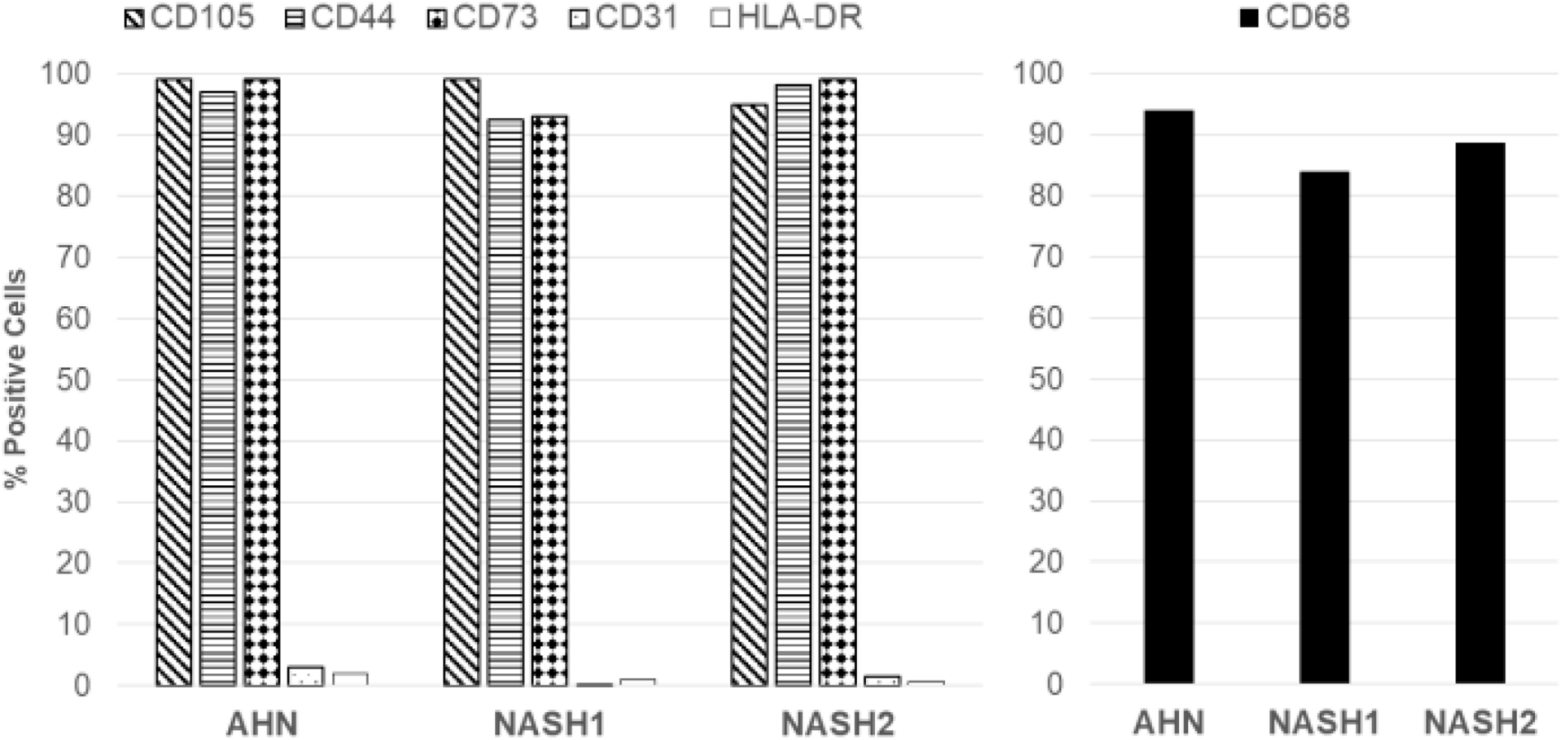
**Generation of MSC (hepatic stellate cell precursor) and macrophages (Kupffer cell analogues).** Quantification of end stage MSC lineage markers CD105, CD44, and CD73, leukocyte/platelet surface marker CD31, and leukocyte surface marker HLA-DR (left) expression by flow cytometry. Quantification of CD68 expression in end-stage macrophage (Kupffer cells) derived from ANH and NASH specific iPSCs by flow cytometry (right).

**Fig. 8.**
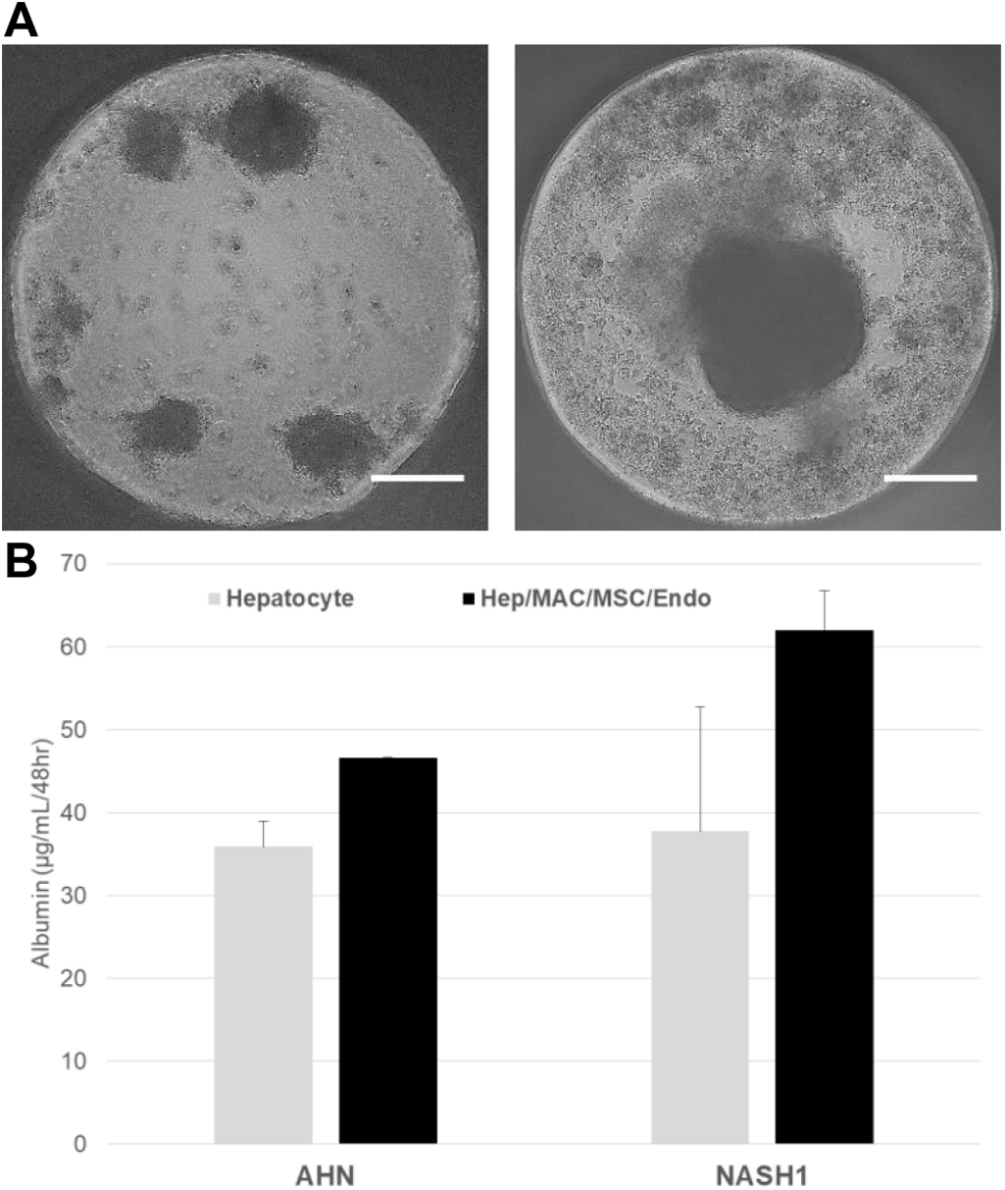
**Morphology and albumin secretion in liver organoids.** (A) A phase contrast image of aggregates consisting of hepatocytes (left) or hepatocytes, macrophages, MSC, and endothelial cells (right) 1 week after aggregate formation and culture in Stage 3 media. Scale bars: 200 µm. (B) Quantification of albumin secretion in aggregates consisting of hepatocytes alone or hepatocytes, macrophages, MSC, and endothelial cells (Hep/MAC/MSC/Endo) measured on day 10 after aggregate formation. The graph denotes average values±s.e. from three biological replicates per condition.

The ability of the hepatocytes to survive and function in a co-culture model was evaluated in the presence of isogenic iPSC derived mesenchymal stem cells (MSC, precursors of hepatic stellate cells), macrophages (Kupffer cell analogues), and endothelial cells from normal and NASH specific iPSCs. The isogenic cell types were derived using protocols used to generate highly pure populations of mesenchymal stem cells, macrophages, and endothelial cells (Fig. 7). Hepatocytes used in the co-culture studies were recovered from end of Stage 1 cryopreservation and the non-parenchymal cell types used for the co-culture studies were recovered from cryopreservation and adapted to Stage 3 media. At the end of Stage 2, hepatocytes were placed in a three-dimensional (3D) co-culture with isogenic stellate-like and Kupffer-like cells at physiologically relevant ratios. The organoid cultures generated from AHN and NASH specific iPSC remained intact and alive for 10 days (Fig. 8A) and maintained hepatic functionality by secreting albumin at higher levels compared to hepatocyte monoculture aggregates (Fig. 8B).

### End-stage hepatocytes manifest a NASH phenotype *in vitro*

**Fig. 9.**
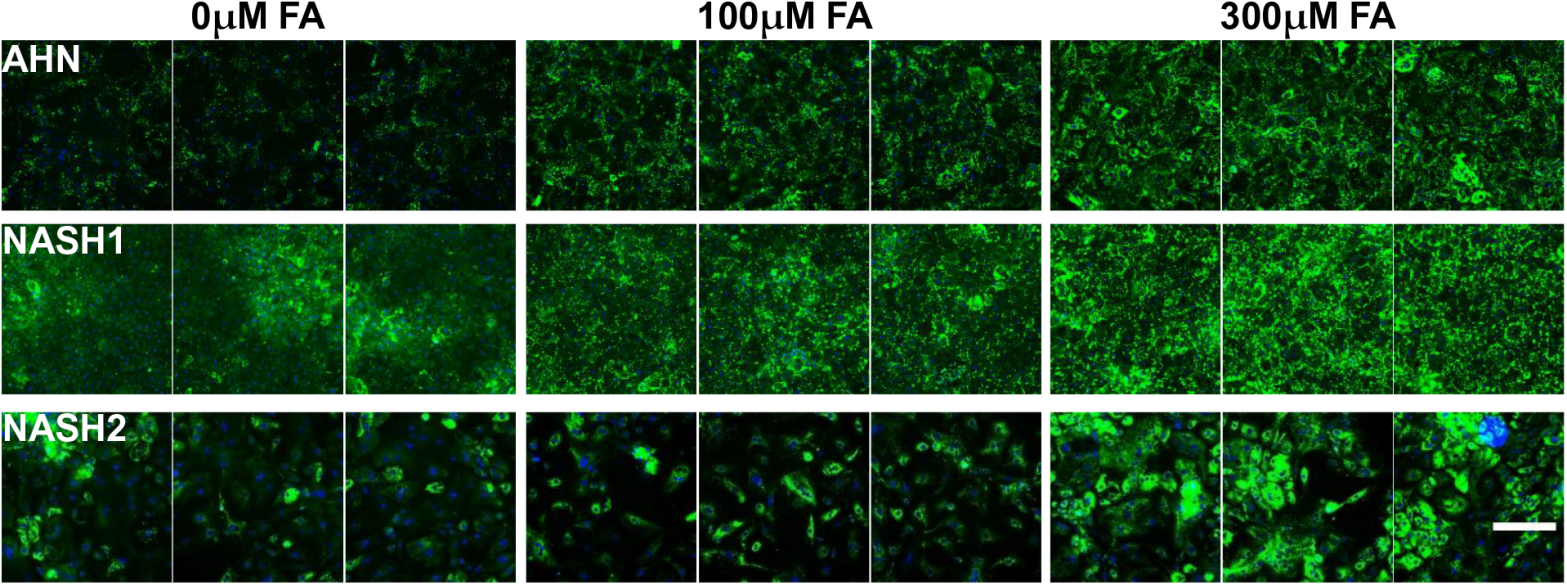
**Intracellular lipid accumulation in hepatocytes at the end of Stage 3.** Visualization of lipid droplets stained by Bodipy (green) and nuclei stained by DAPI (blue) in end stage hepatocytes derived from ANH and NASH1 and NASH 2 specific iPSCs. Scale bar: 50 µm.

Excessive lipid accumulation is a hallmark of NAFLD and NASH. Several cell culture based models of NAFLD/NASH have been described recently, including those using iPSC derived hepatocytes, where a NASH-like phenotype was induced by exposure of the cells to increased lipid levels (Parafati et al., 2018). Evaluation of iPSC derived end stage hepatocytes as a model of NAFLD/NASH was performed by quantifying lipidosis post FA supplementation. End stage hepatocytes derived from both AHN and NASH donors displayed a dose-dependent increase in intracellular lipid accumulation when the cells were exposed to a combination of oleic and linoleic acids. Interestingly, hepatocytes derived from NASH donors displayed the spontaneous accumulation of extracellular lipids in the absence of exogenous FA supplementation, while hepatocytes from AHN donor did not (Fig. 9). Thus, hepatocytes differentiated from NASH donors successfully preserved and recapitulated steatosis, one of the key features of the fatty liver disease under *in vitro* conditions.

## DISCUSSION

We have developed and validated a novel differentiation protocol for deriving hepatocyte-like cells from iPSCs from both AHN donors and NASH donors. The hepatocytes produced by this protocol displayed the hallmark phenotypic features of hepatocytes: high purity based on the expression of hepatic markers such as HNF4α, AAT, ASGR1 and albumin, CYP3A4 activity induction in response to rifampicin, typical hepatic morphology when plated onto collagen, and formation of bile canaliculi (Figs 4 and 5). Based on the ratios of *CYP3A4* to *CYP3A7* and P1 to P2 transcripts of *HNF4A* (Fig. 5), the cells produced by this protocol are intermediate in their maturation level falling between fetal and adult hepatocytes.

GSK3 inhibitor CHIR99021 supplementation enhanced the purity DE cultures and enhanced expansion/yield of cultures during Stage 2 of differentiation (Fig. 3), thus contributing to an enhanced conversion efficiency of iPSCs to end stage hepatocytes. GSK3 inhibition potentiates canonical Wnt signaling to generate DE from iPSCs (Loh et al., 2014). GSK3 inhibition by CHIR99021 has been shown to promote exit from pluripotency (Teo et al., 2014), which, in turn, has been shown to improve quality of hepatocyte differentiation of hiPSCs (Czysz et al., 2015). CHIR99021 supplementation has also been shown to promote cell proliferation during differentiation of cardiac and neuronal cell types from pluripotent stem cells (Fan et al., 2018; Pachenari et al., 2017), as well as promoting expansion of primary human hepatocytes in 3D cultures (Peng et al., 2018).

The hepatocytes produced by this protocol were readily cryopreservable at intermediate stages of differentiation, and displayed similar properties as non-cryopreserved or fresh hepatocytes (Fig. 6). There are numerous published protocols for hepatic differentiation of human embryonic and pluripotent stem cells (Fig. 1A and Toba et al., 2020), but these lack robustness for performance across donor lines and for the most part yield boutique quantities of cells at the end of the process. The protocol described here routinely produces tens of millions of cryopreserved cells across multiple lines from AHN and NASH donors.

Hepatocytes produced by this protocol, from both AHN and NASH donors, displayed increased lipid accumulation in response to FA exposure (Fig. 9). Interestingly, the hepatocytes from NASH donors exhibited spontaneous lipid accumulation in the absence of FA supplementation mimicking a feature of *in vivo* NASH hepatocytes. This study describes the first *in vitro* differentiation protocol for generating hepatocyte-like cells from NASH iPSCs while preserving the NASH phenotype.

The hepatocytes produced by this protocol were able to successfully integrate into 3D liver organoids with macrophages, MSCs and endothelial cells and these aggregates maintained their hepatic functionality for at least 10 days (Fig. 8). This is of a particular advantage for modeling NAFLD and NASH. Working with animal models for the disease still poses challenges in identifying those best mirroring human pathology (Lau et al., 2017) and while a NASH-like phenotype can be induced in monolayer cultures of primary human hepatocytes and iPSC derived hepatocytes (Parafati et al., 2018), such cultures rapidly decline in their performance owing to spontaneous loss in xenobiotic metabolism capacity and hormone responsiveness (Berger et al., 2015; Mazza et al., 2015). 3D hepatocyte monocultures recapitulate *in vivo* biology more faithfully than monolayer cultures (Sengupta et al., 2014). Hepatic co-cultures have been shown to model NAFLD and NASH more accurately still, and are emerging as the closest system to mimicking the disease *in vitro* (Berger et al., 2015). Importantly, the NASH patient iPSC lines used in this study can be differentiated into analogues of Kupffer cells and hepatic stellate cell precursors (Fig. 7), which together with the hepatocytes described here will provide a useful source of isogenic cells for organoid formation. Lot-to-lot variability of primary human hepatocytes is a recognized issue in their use as a model and to this end, commercial sources of 3D liver organoids typically combine cells from multiple donors (Kaserman and Wilson, 2017; Kermanizadeh, 2019; Kermanizadeh et al., 2019). While such models are very useful in disease modeling and toxicology testing, donor-to-donor variability in the sources of non-parenchymal cells, especially Kupffer cells, negatively affects their performance (Kermanizadeh et al., 2019), and donor matched hepatocyte-Kupffer cell co-cultures display a higher sensitivity in hepatotoxicity studies than donor mismatched ones (Tasnim et al., 2019).

In summary, we developed and tested a novel defined process for producing pure and cryopreservable hepatocytes along with the accessory non-parenchymal cell lineages from episomally reprogrammed iPSCs derived from healthy and NASH donors. These end stage cryopreserved cell types alone or in combination, generated in large quantities, will be an ideal tool set for preclinical evaluation of therapeutic targets for NAFLD/NASH.

## MATERIALS AND METHODS

### Cell lines

AHN iPSCs from donor line 01279 were developed by FUJIFILM Cellular Dynamics, Inc. (https://hpscreg.eu/cell-line/CDIi001-A). Several NASH and AHN iPSCs were purchased from the California Institute for Regenerative Medicine (CIRM) iPSC repository. CIRM donor identifications are listed in Table S1.

### Cell culture

iPSCs from AHN and NASH donors were maintained in Essential 8 (E8) media (Thermo Fisher Scientific, cat. #A1517001) on Matrigel (Corning, cat. #354230). Cells were maintained under hypoxic conditions for at least ten passages and confirmed to have a normal karyotype prior to initialization of hepatocyte differentiation.

To initiate hepatic differentiation, iPSCs were plated at 1.7×10^4^ cells/cm^2^, in E8 with 1μM H1152 onto Matrigel coated vessels. After 2 days of culture in E8 with daily media exchanges, media was changed to preconditioning media containing 3 μM CHIR99021 and cultured for 2 days with daily media exchanges. Definitive endoderm differentiation was then induced with T0 media for 1 day, followed by T1-2 media for 2 days, and then T3-6 media for further 6 days. Hepatic differentiation was induced in three stages: Stage 1 for 6 days, Stage 2 for 8 days, Stage 3 for 7–14 days. Media was exchanged daily during DE induction and then every other day for the remainder of the process (Fig. 1B). Media compositions are given in Table 1. At the end of Stage 1, the cells were detached from vessel surface with Accumax (Innovative Cell Technologies, Inc., cat. #AM105) and seeded at 0.5×10^6^ cells/ml in Stage 2 media +1μM H1152 to form aggregates. Differentiation was carried out under hypoxic conditions until the middle of Stage 2, when the cells were moved to a normoxic incubator.

**Table 1.**
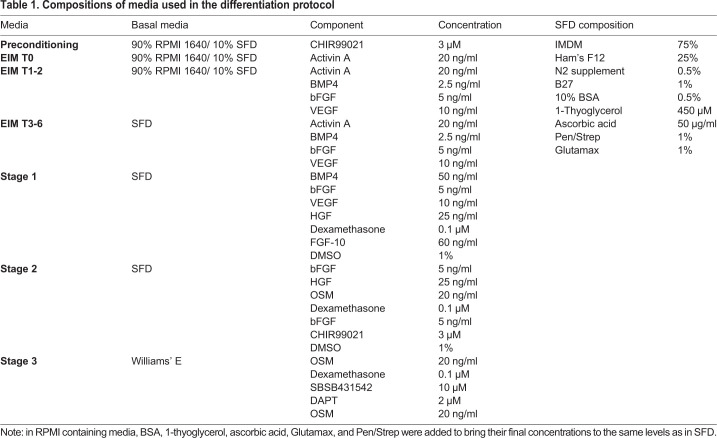
**Compositions of media used in the differentiation protocol**

### Flow cytometry

Cells at different stages of the differentiation process were individualized using TrypLE Select (Thermo Fisher Scientific, cat. #12563) for definitive endoderm analysis or 0.5% Trypsin-EDTA (Thermo Fisher Scientific, cat. #1540054) for later stage staining. For evaluation of definitive endoderm makers, cells were stained live immediately after collection for 30 min at 4°C; whereas for AAT, ASGPR1, or albumin, the cells were fixed with 4% PFA, and stained overnight at 4°C in staining buffer (1 mg/ml Saponin, 2% FBS, in Dulbecco's PBS, Thermo Fisher Scientific, cat. #14190144), followed by a 1-h staining with appropriate AlexaFluor-647 secondary antibody. Cells were analyzed on BD Accuri C6 Plus flow cytometer (BD Biosciences).

### Antibodies

The following primary antibodies were used: PE-conjugated mouse anti-CXCR4 (1:400, BioLegend, cat. #306506), APC-conjugated mouse anti-CD117 (1:500, Thermo Fisher Scientific, #CD117051), FITC-conjugated goat anti-AAT (1:400, Bethyl Laboratories, cat. #A800-122F), mouse anti-albumin (1:5000, Cedarlane, cat. #CL2513A). Secondary antibody used was goat anti-mouse AlexaFluor 647 (1:2000, Thermo Fisher Scientific, #A21240,).

### RNA isolation and qPCR

RNA was isolated using RNeasy kit (Qiagen, cat. #74106) according to the manufacturer’s instructions. Human liver total RNA was purchased from Thermo Fisher Scientific (cat. #AM7960). cDNA was synthesized using Applied Biosystems High Capacity RNA-to-cDNA kit (Thermo Fisher Scientific, cat. #4387406). qPCR was performed using Taqman probes and ABI Taqman Gene Expression Master Mix (Thermo Fisher Scientific) on Roche Light Cycler 480 and analyzed using the Roche Light Cycler 480 software v. 1.5.1.

### Cryopreservation and post-cryopreservation cell recovery

Cells were detached from vessel surfaces using TrypLE Select (Thermo Fisher Scientific, cat. #12563) for end of DE cryopreservation or Accumax (Innovative Cell Technologies, Inc., cat #AM105) for end of Stage 1 cryopreservation, gently dissociated by pipetting and filtered through a 100 μm cell strainer. Cells were then resuspended in Bambanker (Wako, cat. #302-14681) at 5×10^6^ cells/ml for end of DE cryopreservation or 10×10^6^ cells/ml for end of Stage 1 cryopreservation. 1 ml of cell suspension was distributed per cryovial and the cells were frozen in a control rate freezer and stored in liquid nitrogen.

For recovery of cryopreserved cells, vials were thawed in a 37°C water bath for 2–3 min, the cell suspension was transferred to a conical tube containing EIM T3-6 (Table 1, for end of DE cells) or Stage 2 media (Table 1, for end of Stage 1 cells) pre-warmed to 37°C. For end of DE cells, the cells were pelleted, suspended in EIM T3-6 media+1 μM H1152 and plated at 1×10^5^ cells/cm^2^ onto Matrigel coated vessels. The cells were cultured in EIM T3-6 media for 2 days with daily media exchanges. After 2 days, the media was changed to Stage 1 media and the differentiation proceeded as described above. For end of Stage 1 cells, the cells were pelleted and resuspended in Stage 2 media+1μM H1152 at 0.5×10^6^ cells to form aggregates. The differentiation then proceeded as described above.

### CYP3A4 activity assay

On days 5–7 of Stage 3, hepatocyte aggregates were transitioned to William’s E media with Hepatocyte Maintenance Supplement Cocktail B (Thermo Fisher Scientific, cat. #CM4000, without dexamethasone) and either vehicle (0.1% DMSO) or 50 µM rifampicin (Sigma-Aldrich, cat. #R7382) for 3 days with daily media exchanges. At the end of 3 days, the cells were dissociated and distributed into 96-well plates (2.5×10^4^ cell s/well, four to six wells per condition) and subjected to CYP3A4 activity measurement using a luminescent P450-Glo CYP3A4 Assay System (Promega, cat. #V9001) according to the manufacturer's instructions.

### Lipidosis assay

At the end of Stage 2, cells were plated onto Collagen I coated plates (Greiner Bio-One, cat. #655956) and maintained in Stage 3 medium for 4–5 days with media exchanges every other day. Cells were then treated with 0–300 µM FAs (oleic acid-linoleic acid mixture, Sigma-Aldrich, cat. #L9656) diluted in Stage 3 media for 24 h. Cells were washed with DPBS twice and fixed with 4% PFA for 20 min at room temperature (RT). After three washes with DPBS, cells were stained with solution containing 1 µg/ml Biodipy 493/503 (Thermo Fisher Scientific, cat. #D3922), Actin-555 (Molecular Probes, cat. #R37112) and DAPI (Molecular Probes, cat. #R37606) in DPBS with 0.1% Triton-X for 20 min at RT in the dark. Cells were imaged using ImageXpress micro confocal high content imager (Molecular Devices).

### Mesenchymal stem cells, macrophages, and endothelial cells

iCell Mesenchymal Stem Cells (cat. # R1098) and iCell Macrophages (cat. # R1114) were from AHN donor 01279 (FUJIFILM Cellular Dynamics, Inc.). NASH donor CW10202 (CIRM iPSC repository) were differentiated using proprietary differentiation protocols used for the manufacture of iCell Mesenchymal Stem Cells and iCell Macrophages. Endothelial cells from line 01279 were from FUIFILM Cellular Dynamics. Cells were thawed according to the respective cell type iCell User's Guide (https://fujifilmcdi.com/) and adapted to hepatocyte Stage 3 media for 1 week prior to initiation of co-culture experiments.

### Liver organoid formation

Hepatocyte aggregates were dissociated with 0.5% Trypsin-EDTA for 7 min at 37°C. At the same time, macrophages, MSCs, and endothelial cells were dissociated with TrypLE Select for 5–7 min at 37°C. All cells were then suspended to a density of 1×10^6^ cells/ml in hepatocyte Stage 3 media and plated in ultra-low attachment (ULA) plates (Corning, cat. #3471) at the physiologically relevant (Ware et al., 2018; Leite et al., 2016; Tasnim et al., 2019) ratio of 1: 0.5: 2: 0.2 hepatocyte: macrophage: MSC: endothelial cell. Aggregates were maintained for 10 days with media exchanges every other day. Media from the last exchange (days 8–10) was collected and secreted albumin was measured using human albumin ELISA (Thermo Fisher Scientific, cat. #EHALB) according to the manufacturer's instructions.

### Statistical analysis

Differentiation data are presented as mean±s.e. of the mean from three independent experiments. Results in Fig. 3A–C are from the single pivotal experiment aimed at determining the effects of CHIR99021. In all subsequent differentiation runs, CHIR99021 was used during preconditioning (48 h period prior to start of DE induction) and no conditions without CHIR99021 were included.

## Supplementary Material

Supplementary information
