# Supplementary material for: iPSC-derived hepatocytes generated from NASH donors provide a valuable platform for disease modeling and drug discovery
Source: Biol Open. 2020 Dec 16;9(12):bio055087. doi: 10.1242/bio.055087 (PMC7758638; doi:10.1242/bio.055087)
Supplement: Supplementary information [file biolopen-9-055087-s1.pdf]

Supplementary Materials

Table S1

Donor ID	Gender	Disease status
CW10201	Male	NASH
CW10202	Male	NASH
CW10042	Female	NASH
CW10045	Female	NASH
CW10189	Male	NASH
CW10054	Female	AHN
CW10024	Female	AHN

California Institute for Regenerative Medicine (CIRM) iPSC repository cell lines used in this study.
